# Supplementary material for: Collaborative intelligence in AI: Evaluating the performance of a council of AIs on the USMLE
Source: PLOS Digit Health. 2025 Oct 9;4(10):e0000787. doi: 10.1371/journal.pdig.0000787 (PMC12510544; doi:10.1371/journal.pdig.0000787)
Supplement: S2 File — (ZIP) [file pdig.0000787.s002.zip › S2 Data Raw data files containing unprocessed question inputs and LLM outputs including transcripts of deliberations/STEP3_Question-6-1_Type-MC-NJ.docx]

**Facilitator** **[Round 1]:** A 48-year-old man comes to the clinic because of a 10-year history of recurrent, intrusive thoughts that his house will be broken into and damaged by criminals or accidentally destroyed by a fire when he is not home. These thoughts have worsened during the past 2 months. He reports now spending 4 hours daily checking that the doors and windows are closed and locked and that the stove and oven are turned off; he previously spent 2 hours daily doing these tasks. He says he cannot keep a job or leave the house very much because of the amount of time he spends checking these things. He has no other history of serious illness and takes no medications. Physical examination shows no abnormalities. On mental status examination, he has an anxious mood and a sad affect. He is fully oriented. He is not having hallucinations or delusions. The most effective pharmacotherapy for this patient is an agent that targets which of the following neurotransmitters? (A) γ-Aminobutyric acid (B) Dopamine (C) Glutamate (D) Norepinephrine (E) Serotonin

**AI_D:** RESTATING THE QUESTION: The patient, a 48-year-old male, is experiencing persistent thoughts related to fear and destruction that are interrupting his daily life and job. His symptoms have escalated over the past decade to where he now spends a considerable amount of his day checking for potential threats to his home. He has no other known illnesses and doesn't take any medications. The query is regarding the most effective pharmacotherapy for his condition - particularly, which neurotransmitter should be targeted by the drug. Neurotransmitters are chemical messengers in the brain, different ones of which are associated with different functions and mood states. PLANNING: To identify which neurotransmitter is the best target for this patient's treatment, we should recognize that the symptoms are suggestive of Obsessive-Compulsive Disorder (OCD). OCD is a chronic condition characterized by uncontrollable recurring thoughts (obsessions) and behaviors the person feels the urge to repeat over and over (compulsions). Commonly implicated neurotransmitters in OCD are serotonin, dopamine, glutamate, and γ-aminobutyric acid (GABA), but the choice of the best target depends on the commonly associated neurotransmitter dysfunction in OCD. CLINICAL REASONING: Given the patient's symptoms of recurrent, intrusive thoughts and repetitive behaviors, he appears to have OCD, which is often associated with dysregulated serotonin transmission. Treatment typically involves selective serotonin reuptake inhibitors (SSRIs) to increase the availability of serotonin in the synaptic cleft, thereby helping to regulate mood and reduce the symptoms of OCD. Although other neurotransmitters like dopamine, glutamate, and GABA may also play roles in anxiety disorders, serotonin dysregulation is most commonly associated with OCD. Thus, a medication that targets serotonin would likely provide the most benefit for this patient. CONCLUDING: The patient's symptoms as described manifest a classic presentation of OCD. SSRIs which aim to regulate serotonin in the brain, are often used to treat such conditions. As such, it can be postulated this patient's treatment should center on serotonin. RESPONSE: (E) Serotonin

**AI_C:** RESTATING THE QUESTION: The clinical scenario presents a 48-year-old man who has a ten-year history of intrusive thoughts about potential harm to his house when he is not at home. These thoughts have become more frequent and intense recently, and he has developed compulsive behaviors, such as checking doors, windows, stove, and oven, which consume a significant part of his day. His high level of anxiety and compulsiveness hinder his ability to work or leave his home, even though he has no other medical conditions and takes no medication. It's important to note that he doesn't experience hallucinations or delusions. The question is asking which neurotransmitter would be most effectively targeted by pharmacotherapy to alleviate his symptoms. PLANNING: The key points in this question are the recurrent intrusive thoughts and the time-consuming checking behaviors, which are causing significant distress and impairment in the patient's life. These are typical symptoms of Obsessive-Compulsive Disorder (OCD), a psychiatric disorder characterized by obsession (recurrent and persistent thoughts) and compulsion (repetitive behavior). Pharmacologically, this condition is thought to be influenced by an imbalance in neurotransmitters like serotonin, dopamine, etc. It's crucial to determine which neurotransmitter most likely influences this disease and thus, should be targeted to alleviate the symptoms. CLINICAL REASONING: Obsessive-compulsive disorder (OCD) is a disorder characterized by obsessions (unwanted and intrusive thoughts) and compulsions (repetitive actions performed to relieve the anxiety caused by the obsessions). Neurobiologically, the pathophysiology of OCD has been associated with alterations among several neurotransmitters, including γ-Aminobutyric acid (GABA), dopamine, glutamate, norepinephrine, and serotonin. However, the first-line pharmacotherapy for OCD is selective serotonin reuptake inhibitors (SSRIs) which increase the availability of serotonin in the synapse. This is because deficiency in serotonin neurotransmission is one of the main causes of OCD. SSRIs effectively reduce the symptoms of OCD in many people, though they may take several weeks to show their full effect. CONCLUDING: While there is evidence that multiple neurotransmitters may play a role in obsessive-compulsive disorder (OCD), serotonin is commonly understood to be the primary neurotransmitter involved. Therefore, selective serotonin reuptake inhibitors (SSRIs), which increase serotonin levels in the brain, are generally the most effective pharmacological treatment. RESPONSE: So, the most effective pharmacotherapy for this patient, who likely has obsessive-compulsive disorder (OCD), would be an agent that targets Serotonin (option E).

**AI_A:** This is essentially asking which neurotransmitter is most effectively targeted by pharmacotherapy in treating a 48-year-old man with recurring intrusive thoughts, particularly centered around his home's security, that have escalated over the past couple of months. Such thoughts have taken up so much of his time that his work life and social functioning have been severely affected. It is made clear that he shows no physical abnormalities, is not hallucinating or delusional, and is in a constant state of anxiety and sadness. Neurotransmitters are naturally occurring chemicals that transmit signals across a synapse from one neuron (nerve cell) to another 'target' neuron. They have a role in physical and mental functions, like heart rate, sleep, appetite, mood, and fear. The options given are (A) γ-Aminobutyric acid or GABA, which is primarily responsible for reducing neuronal excitability throughout the nervous system; (B) Dopamine, important for pleasure and reward and also linked to compulsive behavior; (C) Glutamate, crucial for cell-to-cell communication; (D) Norepinephrine that impacts attention and response actions, and (E) Serotonin, a key regulator of mood, appetite, and sleep. Assuming that this man's manifestations are due to an anxiety disorder and considering the impact on his daily functioning, intrusive thoughts, and checking behaviors, it could be surmised that he's dealing with obsessive-compulsive disorder (OCD). OCD is commonly treated with selective serotonin reuptake inhibitors (SSRIs). While other neurotransmitters like GABA and Dopamine may play a role in mood regulation and reward systems, Serotonin's role in mood regulation makes it a leading candidate for treatment. Is it possible that this man's condition could be improved by targeting GABA, Dopamine, Glutamate or Norepinephrine instead of Serotonin? Given that SSRIs are the first-line treatment for OCD and have proven their effectiveness, assuming otherwise might be less likely. The man's symptoms also align with the known function of Serotonin in the brain. We should hypothesize that Serotonin is the neurotransmitter we should focus on for pharmacotherapy. Drawing on what is known about the role Serotonin plays in mood regulation and the efficacy of SSRIs in treating OCD, we can deduce that the best course of treatment would likely involve agents that target Serotonin. This patient's intrusive thoughts, the escalation in obsessive behaviors, and manifesting anxiety and sadness are all symptoms that would likely respond to a manipulation of Serotonin levels in his brain. Considering the known science and empirical evidence regarding the varying roles of different neurotransmitters, the particularly impactful role of Serotonin in mood regulation, and the condition the patient appears to be suffering from - it would seem that targeting Serotonin would be the most effective course of action for this man's pharmacotherapy. Therefore, the response is (E) Serotonin. The SSRIs, which are one of the first-line treatments for OCD, work by increasing the level of Serotonin in the brain, which in turn can help reduce obsessive thoughts and compulsive behaviors.

**AI_E:** RESTATING THE QUESTION: This query seeks to find out which neurotransmitter-targeting medication would best improve the patient's symptoms. These symptoms include persistent, worsening fears of his home being broken into or unintentionally destroyed, leading him to spend significantly increasing amounts of time double-checking security and utilities. This behavior significantly impacts his ability to maintain employment and generally leave his home. These symptoms, without the presence of delusions or hallucinations, point towards a certain mental health disorder. The suggested neurotransmitters are γ-Aminobutyric acid, dopamine, glutamate, norepinephrine, and serotonin. PLANNING: The main assumptions are: 1) the patient's symptoms suggest a specific psychiatry disorder; and 2) this disorder is best treated within the context of a specific neurotransmitter dysfunction. The patient's described fears could point towards an anxiety disorder, such as OCD characterized by persistent thoughts (obsessions) that lead to repetitive behaviors (compulsions). Now, the dysfunction of which neurotransmitter is most typically associated with OCD? γ-Aminobutyric acid (GABA) is the main inhibitory neurotransmitter in the brain and an increase in GABA activity is associated with anti-anxiety effects. Dopamine imbalance may play a role in feeling distracted or unable to focus, which are symptoms associated with ADHD. However, his symptoms seem less related to dopamine dysfunction. Glutamate is the main excitatory neurotransmitter in the nervous system and might be involved in the pathophysiology of various psychiatric disorders including OCD, but its precise role is still being researched. Norepinephrine dysfunction could lead to mood disorders such as depression or anxiety but that does not seem to be the primary issue here. Serotonin imbalance, however, is closely linked to depression and OCD. CLINICAL REASONING: The patient displays classic signs of obsessive-compulsive disorder (OCD), a mental disorder where people have recurring, unwanted thoughts, ideas or sensations (obsessions) that make them feel driven to do something repetitively (compulsions). This often results in the person spending a lot of time on these obsessions, which can interfere with daily activities including employment. The main underlying etiology of OCD has been suggested to be a dysfunction within the serotonergic system. More specifically, it is thought that there is a decrease in serotonin activity. This is why SSRIs (selective serotonin reuptake inhibitors), which increase the extracellular level of serotonin by limiting its reabsorption into the presynaptic cell, are often used as a first-line treatment in OCD. CONCLUDING: This patient demonstrates characteristics suggesting obsessive-compulsive disorder—a condition that has been associated primarily with disrupted serotonergic neurotransmission. In context, the most likely relevant neurotransmitter involved seems to be serotonin. RESPONSE: (E) Serotonin

**AI_B:** RESTATING THE QUESTION: This inquiry is about a 48-year-old man who has been dealing with recurrent worries about potential disasters at his residence over the past decade. His fears center on the possibility of criminals breaking into his home or an accidental fire destroying his property. There has been a noticeable escalation in terms of the severity of his anxieties over the last 2 months with him reportedly spending more time checking and ensuring that his home is safe. The patient's state of mind is affecting the quality of his life, particularly with respect to his job continuity and social activities. It's important that he has no past history of significant illnesses and does not use any prescribed drugs. Furthermore, there are no unusual physical findings and his mental status examination reveals an anxious mood plus a sad affect but he is lucid, not hallucinating or delusional. The question is attempting to identify the most appropriate pharmacotherapy for this patient's symptoms, narrowing down the options to neurotransmitter-targeting agents: γ-Aminobutyric acid (GABA), Dopamine, Glutamate, Norepinephrine, and Serotonin. PLANNING: Existing assumptions include that the man's behaviours are a result of a psychiatric condition, that this condition can be effectively managed using pharmacotherapy, and that targeting neurotransmitters with medication is the most favourable approach. We can question each assumption by asking whether the behaviors could be symptomatic of other physical conditions, whether cognitive-behavioral approaches would be more effective, and whether treatments targeting other brain structures or systems might work better. Given the demonstrated clinical efficacy of pharmacological interventions in managing anxiety-related and obsessive-compulsive disorders and the strong evidence linking neurotransmitters to these disorders, the assumption that using neurotransmitter-targeting medication could be valid and worth exploring first. CLINICAL REASONING: Given the patient's longstanding intrusive thoughts and relentless rituals, he likely suffers from Obsessive-Compulsive Disorder (OCD). OCD is characterized by persistent, unwanted thoughts (obsessions), which lead to behaviors that the person feels they must carry out (compulsions). Both obsessions and compulsions can be incredibly time-consuming and debilitating, affecting a person's ability to function at work or maintain a social life. It's also crucial to note that while the man presented is fully aware of the irrationality of his fears and behaviors, he is still unable to control them — a significant trait of OCD. When it comes to pharmacotherapy, SSRIs, which increase the serotonin levels in synapses, have been proven effective in treating OCD. Therefore, a treatment plan that targets serotonin seems to be a reasonable hypothesis to explore. CONCLUDING: Serotonin appears to be the missing link in this clinical puzzle. Selective serotonin reuptake inhibitors (SSRIs) are often the first line of treatment in managing OCD. They work by increasing the level of serotonin, a neurotransmitter involved in mood regulation and anxiety management, among other things. This patient, likely suffering from OCD, would benefit from a treatment plan that targets serotonin. RESPONSE: The most effective pharmacotherapy for this patient, who presents with symptoms that align with Obsessive Compulsive Disorder, is an agent that targets Serotonin (E) neurotransmitter.

**Facilitator** The question presents a 48-year-old man who has been experiencing recurrent, intrusive thoughts about potential harm to his home for the past decade. These thoughts have escalated in intensity and frequency over the past two months, leading to compulsive behaviors such as repeatedly checking doors, windows, and appliances. These behaviors consume a significant portion of his day, affecting his ability to maintain employment and engage in social activities. The man is not hallucinating or delusional, and he exhibits an anxious mood and a sad affect. The question asks which neurotransmitter should be targeted by pharmacotherapy to most effectively treat this patient's symptoms. The symptoms described suggest that the man is likely suffering from Obsessive-Compulsive Disorder (OCD), a psychiatric condition characterized by persistent, unwanted thoughts (obsessions) and behaviors that the person feels compelled to repeat (compulsions). These obsessions and compulsions can be time-consuming and debilitating, affecting a person's ability to function in daily life. Neurobiologically, the pathophysiology of OCD has been associated with alterations among several neurotransmitters, including γ-Aminobutyric acid (GABA), dopamine, glutamate, norepinephrine, and serotonin. However, the first-line pharmacotherapy for OCD is selective serotonin reuptake inhibitors (SSRIs), which increase the availability of serotonin in the synapse. This is because a deficiency in serotonin neurotransmission is one of the main causes of OCD. SSRIs effectively reduce the symptoms of OCD in many people, though they may take several weeks to show their full effect. While there is evidence that multiple neurotransmitters may play a role in OCD, serotonin is commonly understood to be the primary neurotransmitter involved. Therefore, selective serotonin reuptake inhibitors (SSRIs), which increase serotonin levels in the brain, are generally the most effective pharmacological treatment. In conclusion, the most effective pharmacotherapy for this patient, who likely has obsessive-compulsive disorder (OCD), would be an agent that targets Serotonin (option E). This conclusion is based on the known role of serotonin in mood regulation, the efficacy of SSRIs in treating OCD, and the patient's symptoms, which align with the known function of serotonin in the brain.
